# Paving the way for integrating genetic testing into clinical care in childhood‐onset schizophrenia

**DOI:** 10.1002/gps3.70057

**Published:** 2026-09-07

**Authors:** Arnaud Fernandez, Philippe Auby, Boris Chaumette, Gaelle Laure, Susanne Thümmler, Florence Askenazy

**Affiliations:** ^1^ CoBTeK (Cognition‐Behaviour‐Technology) Lab Université Côte d’Azur Nice France; ^2^ Centre de Référence pour les Maladies Rares à expression psychiatrique (PsyRare) Nice France; ^3^ University Department of Child and Adolescent Psychiatry, Children's Hospitals of NICE CHU‐Lenval Nice France; ^4^ Université Paris Cité, NeuroDiderot (INSERM U1141), Institut Pasteur (CNRS UMR3571), GHU Paris Psychiatrie et Neurosciences Paris France; ^5^ Department of Psychiatry McGill University Montreal Canada

Childhood‐onset schizophrenia (COS) is a rare (1/10 000) and severe disorder starting before the age of 13 and characterised by substantially higher rates of neurocognitive impairment and pathogenic genetic variants than adult‐onset schizophrenia (AOS).^1^, ^2^ This biological severity is mirrored by profound clinical consequences. Hence, COS is frequently associated with a poorer long‐term prognosis, higher rates of treatment resistance and a profound disruption of social and academic trajectories, resulting in a major burden for patients, their families and society.^2^

Although genetic testing has been integrated into clinical care in other medical conditions when they have an early onset, such as breast cancer and Alzheimer's disease, to allow for precision medicine, this is not yet standard practice in child psychiatry. Likewise, a significant gap exists between the genetic reality of COS and the major international diagnostic and treatment guidelines, which do not currently endorse routine genetic testing for COS unless it is accompanied by autism spectrum disorder (ASD), intellectual disability (ID) or syndromic features^3^, ^4^ (American Academy of Child and Adolescent Psychiatry, American College of Medical Genetics and Genomics, and Royal College of Psychiatrists).

We argue that this restrictive framework, although historically cautious, is now scientifically outdated. Arguments against broader testing across the schizophrenia spectrum often cite the modest diagnostic yield (∼6%). Specifically, ID was significantly associated with increased diagnostic yield (estimate = 0.0457, *p* = 0.001), whereas early age of onset showed a promising but nonsignificant statistical trend (estimate = 0.0348, *p* = 0.089).^5^ However, this figure primarily originated from unselected AOS cases. It is well established that, in COS, the diagnostic yield is considerably higher, ranging from more than 10% in classical chromosomal microarray (CMA) studies to 19% in a recent exome sequencing cohort.^2^, ^3^, ^4^, ^5^, ^6^ In addition, even with a broader age‐of‐onset criterion (< 18 years), the burden of copy‐number variants (CNVs) was comparable to that observed in ASD, independently of autistic comorbidity.^7^ By limiting testing to severe neurodevelopmental or syndromic cases, current practice creates a self‐reinforcing dynamic: Children presenting with COS but without ID or associated syndromic features are excluded from genomic sampling, which, in turn, maintains the uncertainty regarding the clinical utility of testing in this group. Consequently, the absence of genetic testing limits our understanding of the variable expression of genetic abnormalities because many untested COS cases may carry the same pathogenic variants identified in individuals with ASD or ID without presenting the same phenotypic features, a phenomenon frequently observed in neurodevelopmental disorders.^8^

The mounting evidence of clinical and genetic overlap between schizophrenia and neurodevelopmental disorders makes this distinction even more artificial^1^, ^2^, ^7^ and provides additional arguments in favour of extending genetic assessments beyond individuals with schizophrenia and co‐occurring ID and/or syndromic features. Intellectual ability is an imperfect criterion, as it is strongly influenced by educational and sociocultural factors. For instance, in 22q11.2 deletion syndrome and probably in other genetic conditions, carriers' intelligence quotients (IQ) are influenced by parental IQs.^9^ Consequently, restricting genetic testing to individuals with ID may exclude patients who have more highly educated parents and who may, despite carrying a pathogenic variant, score within the normal IQ range.

Our translational research within the GenAuDiss protocol,^10^, ^11^ designed to explore the prototypical overlap between COS and autistic features, provides initial data consistent with the high heterogeneity of these conditions. Of the 20 children with nonsyndromic COS, all of whom underwent initial CMA, pathogenic CNVs were identified in 10% (2/20). Exome sequencing was subsequently performed in CMA‐negative cases and completed in 10 patients at the time of analysis, identifying a likely pathogenic single‐nucleotide variant in 10% (1/10). Overall, the sequential testing strategy yielded a molecular diagnosis in 15% (3/20) of the nonsyndromic cohort.^11^ Although this stepwise approach (CMA followed by exome sequencing), driven by cost and access constraints, aligns with historical algorithms, we now advocate first‐tier whole‐genome sequencing (WGS) as the optimal standardised pathway. WGS provides a comprehensive genomic assessment in a single test, detecting virtually all clinically relevant CNVs detectable by CMA, as well as smaller CNVs and other classes of genetic variation. This single‐tier approach maximises diagnostic yield while reducing the diagnostic odyssey and shortening the turnaround time to a molecular diagnosis.

Of course, local implementation will inevitably depend on regional resources and reimbursement policies, but the systematic integration of genetic testing into routine clinical care has the potential to bridge the gap between academic research and clinical practice. By moving beyond restricted research cohorts, this approach addresses the sampling bias that has historically left the genetics of many children with COS unexamined. However, evaluating the true value of this approach requires a clear distinction between diagnostic yield and the broader components of clinical utility. Clinical experience already points to several meaningful outcomes.

First, identification of a rare genetic disorder can offer immediate benefits, most notably through psychiatric genetic counselling to support families in understanding heritability and risk.^12^

Second, most genetic variants are not directly actionable, with notable exceptions such as neurometabolic disorders. However, several examples support improved clinical management after genetic testing, including evaluation of potential comorbidities, prevention of side effects and use of pharmacogenetics to optimise medication selection and dosing, thereby potentially improving tolerability and effectiveness. Although genetics‐informed disease modelling and personalised treatments hold significant promise for the future, their current translation into clinical outcomes remains a complex and long‐term challenge.^12^, ^13^

Third, the cost‐effectiveness of this approach must be evaluated by balancing upfront sequencing costs against the systemic benefits of shortening the diagnostic odyssey. In France, the structural framework required to implement this approach is already integrated into the national genomic programme (Plan France Médecine Génomique),^14^ which facilitates equitable access to systematic genetic testing for the full spectrum of patients with COS and other complex psychiatric disorders.

Finally, potential harms must also be considered, including psychological distress, genetic stigma and clinical uncertainty associated with variants of uncertain significance. However, these challenges are not unique to COS and are already routinely addressed when offering WGS to individuals with ASD or ID.

Given the growing evidence that COS lies within the neurodevelopmental continuum, there is little rationale for maintaining a different diagnostic paradigm. We therefore believe that the current evidence justifies broad access to clinical genetic testing for all individuals with COS. Although implementing first‐tier WGS as a routine standard of care will require further evidence of clinical utility and cost‐effectiveness, we believe that it represents the most promising diagnostic pathway for COS and has the potential to become the standard of care.

## AUTHOR CONTRIBUTIONS

Arnaud Fernandez performed the literature search, analysed the data and drafted the original manuscript. Gaelle Laure, Boris Chaumette and Philippe Auby provided specific expertise in genetics and clinical psychiatry. Susanne Thümmler and Florence Askenazy provided senior supervision and critical institutional resources, and revised the manuscript for important intellectual content. All authors (Arnaud Fernandez, Philippe Auby, Boris Chaumette, Gaelle Laure, Susanne Thümmler, Florence Askenazy) reviewed and approved the final version of the manuscript for submission.

## FUNDING

Boris Chaumette was supported by the Inserm‐AAP Messidore 2022 grant (No. 9), funded by Inserm and the French Ministry of Health, to support the implementation of whole‐genome sequencing in routine clinical practice in France.

## CONFLICT OF INTEREST STATEMENT

The authors declare no conflicts of interest.

## ETHICS STATEMENT

This commentary discusses findings from the GenAuDiss protocol, which was previously approved by the relevant Institutional Review Board (reference 11). All clinical procedures were conducted in accordance with the Declaration of Helsinki, and informed consent was obtained from all participants or their legal guardians during the original study.

## Data Availability

The data that support the findings of this study are available from the corresponding author, Arnaud Fernandez, upon reasonable request.
